# MicroRNA‐582‐5p targeting Creb1 modulates apoptosis in cardiomyocytes hypoxia/reperfusion‐induced injury

**DOI:** 10.1002/iid3.708

**Published:** 2022-10-11

**Authors:** Rui‐Ze Niu, Lu‐Qiao Wang, Wei Yang, Li‐Zhong Sun, Jie Tao, Huang Sun, Song Mei, Wen‐Jie Wang, Ke‐Xiang Feng, Dian‐Lun Qian, Xiang‐Feng Bai

**Affiliations:** ^1^ Department of Cardiac Surgery Kunming Medical University First Affiliated Hospital Kunming Yunnan China; ^2^ Department of Animal Zoology Kunming Medical University Kunming Yunnan China; ^3^ Department of Cardiology Kunming Medical University First Affiliated Hospital Kunming Yunnan China; ^4^ Department of Anesthesiology Kunming Medical University First Affiliated Hospital Kunming Yunnan China; ^5^ Department of Cardiovascular Surgery, Beijing Anzhen Hospital, Beijing Institute of Heart, Lung and Blood Vessel Diseases Capital Medical University Beijing China

**Keywords:** apoptosis, Creb1, miR‐582‐5p, myocardial ischemia–reperfusion injury

## Abstract

**Background:**

Myocardial ischemia–reperfusion injury (MIRI) caused by the reperfusion therapy of myocardial ischemic diseases is a kind of major disease that threatens human health and lives severely. There are lacking of effective therapeutic measures for MIRI. MicroRNAs (miRNAs) are abundant in mammalian species and play a critical role in the initiation, promotion, and progression of MIRI. However, the biological role and molecular mechanism of miRNAs in MIRI are not entirely clear.

**Methods:**

We used bioinformatics analysis to uncover the significantly different miRNA by analyzing transcriptome sequencing data from myocardial tissue in the mouse MIRI model. Multiple miRNA‐related databases, including miRdb, PicTar, and TargetScan were used to forecast the downstream target genes of the differentially expressed miRNA. Then, the experimental models, including male C57BL/6J mice and HL‐1 cell line, were used for subsequent experiments including quantitative real‐time polymerase chain reaction analysis, western blot analysis, hematoxylin and eosin staining, flow cytometry, luciferase assay, gene interference, and overexpression.

**Results:**

MiR‐582‐5p was found to be differentially upregulated from the transcriptome sequencing data. The elevated levels of miR‐582‐5p were verified in MIRI mice and hypoxia/reperfusion (H/R)‐induced HL‐1 cells. Functional experiments revealed that miR‐582‐5p promoted apoptosis of H/R‐induced HL‐1 cells via downregulating cAMP‐response element‐binding protein 1 (Creb1). The inhibiting action of miR‐582‐5p inhibitor on H/R‐induced apoptosis was partially reversed after Creb1 interference.

**Conclusions:**

Collectively, the research findings reported that upregulation of miR‐582‐5p promoted H/R‐induced cardiomyocyte apoptosis by inhibiting Creb1. The potential diagnostic and therapeutic strategies targeting miR‐582‐5p and Creb1 could be beneficial for the MIRI treatment.

## Graphical abstract

We used bioinformatics analysis to find significantly different microRNA (miRNA) by analyzing transcriptome sequencing data from myocardial tissue in the mouse myocardial ischemia–reperfusion injury (MIRI) model. Multiple miRNA‐related databases (miRdb, PicTar, and TargetScan) were used to predict the downstream target genes of miRNA. Finally, we demonstrated that upregulation of miR‐582‐5p promotes apoptosis induced by hypoxia/reperfusion (H/R) in HL‐1 cells by inhibiting cAMP‐response element‐binding protein 1 (Creb1). Therapeutic strategies that target miR‐582‐5p and Creb1 could be beneficial for the treatment of ischemia/reperfusion injury after acute myocardial infarction.

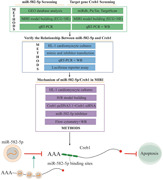

## 1 INTRODUCTION

Coronary heart disease (CHD) is one of the common and high‐risk diseases and remains an important cause of disability and death.^1^, ^2^ Acute myocardial infarction (AMI) and myocardial ischemia–reperfusion injury (MIRI) have been recognized as the principal cause of CHD and subsequent long‐term complications.^3^, ^4^ Rapid recovery of blood flow in the ischemic myocardium is critical to the treatment of AMI. However, along with this restoration of blood flow, additional complications and further cardiomyocyte death gradually develop, termed MIRI.^2^ MIRI is a pathological phenomenon induced by an imbalance in blood supply and demand within the ischemic tissue/organ, commonly characterized by severe histanoxia and microvascular dysfunction. In the case of complete or near restoration of blood supply, the reperfusion can strengthen the activation of immune responses and various cell death programs.^5^ To date, the pathological mechanisms underlying MIRI development remain ambiguous. Apoptosis and necrosis of cardiomyocytes were reported to be two important ways to cause cardiomyocyte damage.^6^ Therefore, further intensive investigation into the pathological mechanisms of MIRI can provide a solid theoretical basis for therapeutic strategies exploration.

Recently, the role of the noncoding RNA (ncRNAs) represented by the microRNA (miRNA) in the regulation of gene expression has been increasingly valued, allowing us to understand gene expression regulation at a new level. It allows us to seek a deeper understanding of the development of the diseases and the mechanism of drug action. Overall, messenger RNA (mRNA) is a template for protein biosynthesis, and that mRNA stability directly affects the number of final products of gene expression, which is also an important factor in the posttranscriptional regulation of gene expression.^7^ As an endogenous small RNA of 20–24 nucleotides long, miRNAs are widely distributed in eukaryotic cells with highly conserved, timing expression and tissue expression specificity.^8^ The posttranscriptional miRNAs generally suppress translation and protein synthesis by base‐pairing to mRNA 3′‐untranslated region (3′‐UTR).^9^, ^10^ More and more research have reported that miRNAs were involved in and regulated many important biological processes in the human body, such as growth and development, apoptosis, cell proliferation, angiogenesis, and so forth.^11^, ^12^ In recent years, the continuous innovation and development of new sequencing technologies enables their wide application in all aspects of biological medicine, promoting the human understanding of the normal physiological function of the body and the occurrence of diseases. Currently, there are more than 5000 sequencing articles on heart tissue and more than 500 sequencing articles on myocardial infarction.^13^, ^14^ Through gene sequencing and comparative analysis, we can easily derive the genes possibly associated with the disease, and subsequently, diagnose or treat the disease according to these genes. Given the difficulty to obtain myocardial tissue in patients with MIRI, the current sequencing of MIRI mainly focuses on experimental animal models.

MiR‐582‐5p is a newly discovered noncoding gene, whose functions are similar to tumor suppressor genes. In recent studies, miR‐582‐5p showed low expression in multiple cancer.^15^, ^16^, ^17^ Increasing miR‐582‐5p expression can significantly inhibit the malignant biological behaviors of tumor cells, including proliferation, invasion, and metastasis. Currently, two studies have reported a possible role of miR‐582‐5p in ischemia–reperfusion injury disease. One of these studies showed that miR‐582‐5p is involved in the ischemia–reperfusion injury after liver transplantation by regulating cell apoptosis.^18^ Another study showed that miR‐582‐5p is involved in the regulation of neural cell apoptosis after cerebral ischemia–reperfusion injury.^19^ These studies suggest that miR‐582‐5p plays an important role in the regulation of cardiomyocyte apoptosis after MIRI. However, the functions of miR‐582‐5p and its underlying mechanisms in MIRI remain uncertain.

CAMP‐response element‐binding protein 1 (Creb1) encodes a transcription factor that binds as a homodimer to the cAMP‐responsive element. The phosphorylated status of Creb1 can induce gene transcription to respond to hormonal stimulation of the cAMP pathway.^20^ In ischemia‐related studies, Creb1 correlated with the ischemic injury and ischemia–reperfusion injury process in various tissues. For example, in the mouse hindlimb muscle ischemic tissue, JNK3 deletion is involved in the upregulated vascular remodeling response during peripheral ischemia through the activation of Creb1, leading to the upregulation of various growth factors in the ischemic muscles.^21^ In cerebral ischemia, Creb1 is recognized as a key transcription factor in the cerebral ischemia response.^22^, ^23^ In AMI, miR‐134‐5p and miR‐662 can regulate cardiomyocyte apoptosis through the regulation of Creb1.^24^, ^25^ Furthermore, studies demonstrated that the expression of phosphorylated Creb1 was significantly associated with the degree of retinal ischemia–reperfusion injury in rats.^26^ These studies showed that Creb1 is mediated in cell apoptosis. However, the functions of Creb1 in MIRI remain obscure.

In the present study, our findings showed that the expression of miR‐582‐5p is significantly increased in hypoxia/reperfusion (H/R)‐induced cardiomyocytes and in MIRI mice, and engaged in H/R‐induced cell apoptosis. In addition, Creb1 was found involved in the stimulative role of miR‐582‐5p in H/R‐induced apoptosis. This study elucidated that miR‐582‐5p/Creb1 axis can potentially serve as diagnostic and therapeutic target for the MIRI.

## 2 MATERIALS AND METHODS

### 2.1 Analysis of transcriptome data

To search for differential miRNAs associated with MIRI, a miRNA sequencing dataset associated with MIRI was downloaded from the Gene Expression Omnibus (GEO): GSE124176 (containing six myocardial tissue samples from three sham and three MIRI).^27^ The miRNAs data were filtered and integrated (considered as differently expressed miRNAs at log‐fold change >2 and adjusted *p* < .05 based on differential fold intensity and relative expression probability), and the data were analyzed, screened, and graphed using the R language program (https://www.r-project.org/).

### 2.2 Predicting the target genes of miRNA

To further obtain the downstream target genes of miRNA, we predicted the target genes of miRNA through the miRdb (http://mirdb.org/),^28^ PicTar (https://pictar.mdc-berlin.de/),^29^ and TargetScan (http://www.targetscan.org/vert_72/) databases.^29^

### 2.3 Animals care

Three‐month‐old male C57BL/6J mice were purchased from the Laboratory Animal Centre of Kunming Medical University. As previously described,^30^ animals were housed individually for pre‐ and postoperative monitoring under a standard light/dark cycle. The Animal Care & Welfare Committee of Kunming Medical University legally approved all animal studies. All experiment procedures were in compliance with the Guide for the Care and Use of Laboratory Animals. Animals were randomly arranged into two groups (*n* = 3/group): MIRI group and Sham group.

### 2.4 MIRI model establishment

Isoflurane was used for continuous respiratory anesthesia. Whole‐course gas anesthesia was performed with a respiratory anesthesia machine (Cat: R510‐22‐16, RWD). Mice were fixed in the supine position and their head and legs were tapes attached to a foam plate for complete exposure to the chest. Mouse chest and axillary hair were removed with hair removal cream (fully exposed to the surgical area), and the surgical area was disinfected with iodophor. Mice were subjected to tracheal intubation along the glottis (tracheotomy: the neck skin of mice was incised, separated layer by layer; the exposed trachea was opened and tightened after intubation). The ventilator parameters were adjusted to a frequency of 120 times/min and a tidal volume of 6–8 ml/kg. A 1.5 cm incision was made at the third and fourth intercostal positions. The skin, thoracic large muscles, and the anterior saw muscle are blunt separated (gently separated using the tip of the round and blunt tweezers). As previously described,^31^ heart was gently squeezed between the third and fourth ribs and exposed to the thorax. Then, the anterior descending artery was ligated to completely block its blood flow. After ligation, the heart tip turns white, and the ligation line was released 30 min later. The whole blood and myocardial tissues were removed after 1 h of reperfusion.

### 2.5 Tissues harvest

All experimental animals were anesthetized with isoflurane for 24 h after reperfusion. After transcardiac perfusion with a precooled 0.01 M phosphate buffer saline (PBS), the whole heart tissue was taken for quantitative real‐time polymerase chain reaction (qRT‐PCR) and western blot (WB) experiments. After transcardiac perfusion with precooled 0.01 M PBS and 4% paraformaldehyde (PFA), hearts were removed in 4% PFA for 24 h and used to prepare paraffin‐embedded tissue sections and staining.

### 2.6 Hematoxylin and eosin staining

Heart tissues were fixed overnight in 4% PFA at 4°C, and then trimmed appropriately followed by sequentially tissue dehydration, paraffin embedding, microtome sectioning, and standard hematoxylin and eosin (HE) staining as described by the Pathology Clinical Service Center of Kunming Medical University.^32^ The images were independently analyzed by an attending pathologist.

### 2.7 HL‐1 cell culture

The HL‐1 cell line used in this study was obtained from the Procell Life Science & Technology Co., Ltd. (Cat: CL‐0605). As previously described, the HL‐1 cardiomyocytes were maintained in high glucose Dulbecco's modified Eagle's medium (DMEM; Cat: 11965092; Gibco) with 10% fetal bovine serum (FBS; Cat: 10099141C; Gibco) and 100 mg/ml penicillin/streptomycin (Cat: 10378016; Gibco).^34^

### 2.8 H/R in HL‐1 cells

Oxygen–glucose deprivation (OGD)/recovery test was performed to establish the H/R model of HL‐1.^34^, ^35^, ^36^ HL‐1 cells were placed in six‐well plates with glucose and serum‐free DMEM and subsequently placed in 37°C hypoxia chambers with 0.5% O_2_, 94.5% N_2_, and 5% CO_2_ for 2 h. After OGD treatment, glucose and 10% FBS were added to plates, and the cells were maintained under a complete medium for an additional 24 h.

### 2.9 Quantitative real‐time polymerase chain reaction

Cultured cells or frozen cardiac muscle tissue were lysed using RNAiso Plus (Cat: 9109; Takara Bio Inc.) for total RNA extraction and then reversely transcribed using Bestar^TM^ qPCR RT Kit (Cat: DBI‐2220; DBI Bioscience) to obtain complementary DNA (cDNA).^37^ The cDNA was mixed with SybrGreen (Cat: DBI‐2143; DBI Bioscience) and primers according to the manufacturer's instructions for qRT‐PCR reactions. The gene expression was normalized to that of GAPDH using the $2^{‐∆∆C_{t}}$ method.^39^ Primers used for the research are as follows:

CREB1‐forward (F) CTGGAGTTGTTATGGCGTCC, CREB1‐reverse (R) TACGACATTCTCTTGCTGCCT; mmu‐miR‐582‐5p‐F CGGCGCATACAGTTGTTCAAC, mmu‐miR‐582‐5p‐R ACTGCAGGGTCCGAGGTATT.

### 2.10 Protein extraction and  WB analysis

Cultured cells or frozen cardiac muscle tissues were lysed using radioimmunoprecipitation assay buffer (Cat: P0013C; Beyotime) to obtain total protein.^39^ Then, the proteins were separated on an sodium dodecyl sulphate‐polyacrylamide gel electrophoresis (Cat: P0012AC; Beyotime) and were transferred to a Hybond‐PVDF membrane (Cat: FFP36; Beyotime). Next, membranes were blocked in 5% milk for 2 h at room temperature (RT) and incubated with primary antibodies overnight at 4°C. The primary antibodies contain Creb1 (rabbit, 1:500, ab32515), Bax (rabbit, 1:1000, ab32503), Bcl‐2 (rabbit, 1:500, ab182858), cleaved‐caspase‐3 (rabbit, 1:500, ab32042), and β‐actin (rabbit, 1:500, ab7817). Following three times washes using TBST, the membrane was incubated with specific secondary antibodies (goat anti‐rabbit IgG, 1:10,000, ab6721) for 1 h at RT, and was washed with TBST three times. The ChemiDoc XRS Imaging System (BioRad) was used to detect and quantify the specific protein. Grayscale determination was performed and statistical analysis using Fiji.^40^

### 2.11 Cell transfection

The cell transfection procedures were performed as previously described.^41^ MiR‐582‐5p inhibitor, miR‐582‐5p mimic, small interfering RNA (siRNA) against Creb1 (siRNA), and the corresponding negative controls were purchased from RIBOBIO. Lipofectamine 3000 transfection reagent was used for all transfections procedures according to the manufacturerʼs instructions (Cat: L3000015; Invitrogen).^42^ Briefly, HL‐1 cells were seeded into six‐well plates overnight until they reached 50%–60% confluence. Oligonucleotides were mixed into lipofectamine 3000 reagents for 5 min at RT, followed by the addition of the Opti‐MEM to a final concentration of 50 nM. The cells were subjected to OGD after transfection for 24 h. The corresponding oligonucleotides sequences are as follows:

Creb1‐siRNA‐F: 5′‐GGCUAACAAUGGUACGGAUTT‐3′, Creb1‐ siRNA‐R: 5′‐AUCCGUACCAUUGUUAGCCTT‐3′; miR‐582‐5p mimic‐F: 5′‐AUACAGUUGUUCAACCAGUUAC‐3′, miR‐582‐5p mimic‐R: 5′‐AACUGGUUGAACAACUGUAUUU‐3′, miR‐582‐5p inhibitor: 5′‐GUAACUGGUUGAACAACUGUAU‐3′.

### 2.12 Dual‐luciferase reporter assay

As previously described,^43^ the dual‐luciferase reporter assay was performed. Briefly, Creb1 3′‐UTR wild‐type (Creb1‐Wt) and its mutant type (Creb1‐Mut) were constructed into luciferase reporter vector pmir‐GLO (Nanjing Qingke Biotechnology Co., Ltd.). HiPerFect transfection reagent (QIAGEN) was used for all transfection procedures. Creb1‐Wt/Mut and miR‐582‐5p mimic or miR‐NC mimic were transfected into HL‐1 cells. The luciferase activity was measured by an automatic microplate reader (Varioskan LUX; Thermo Fisher Scientific) after 24 h. The ratio of Firefly to Renilla luciferase activity was calculated as the relative luciferase activity.^44^

### 2.13 Flow cytometry

As previously described,^44^ the apoptosis was analyzed by using Annexin V‐fluoresceinlsothiocyanate (FITC)/propidine iodide (PI) Kit (Cat:40302, Beyotime) on flow cytometry (Becton Dickinson). FITC‐Annexin V and PI were used to label apoptotic cells. The fluorescence was analyzed and quadrants were positioned on Annexin V/PI plots to distinguish apoptotic cells on flow cytometry.^44^

### 2.14 Statistical analysis

SPSS 26.0 software was used to analyze all experimental data. The analysis of variances was used for comparing multiple groups and the two‐tailed paired Student's t‐test was used for comparison between two groups. *p* < .05 were considered statistically significant difference, and “*” represents *p* < .05; “**” represents *p* < .01; and “***” represents *p* < .001. Graphs were generated using Graphpad Prism 9, Adobe Photoshop CS6, and Adobe Illustrator 2021.^46^

## 3 RESULTS

### 3.1 Bioinformatics predictions and in vivo MIRI models validate differentially expressed miRNAs and their target genes in MIRI

**Figure 1:**
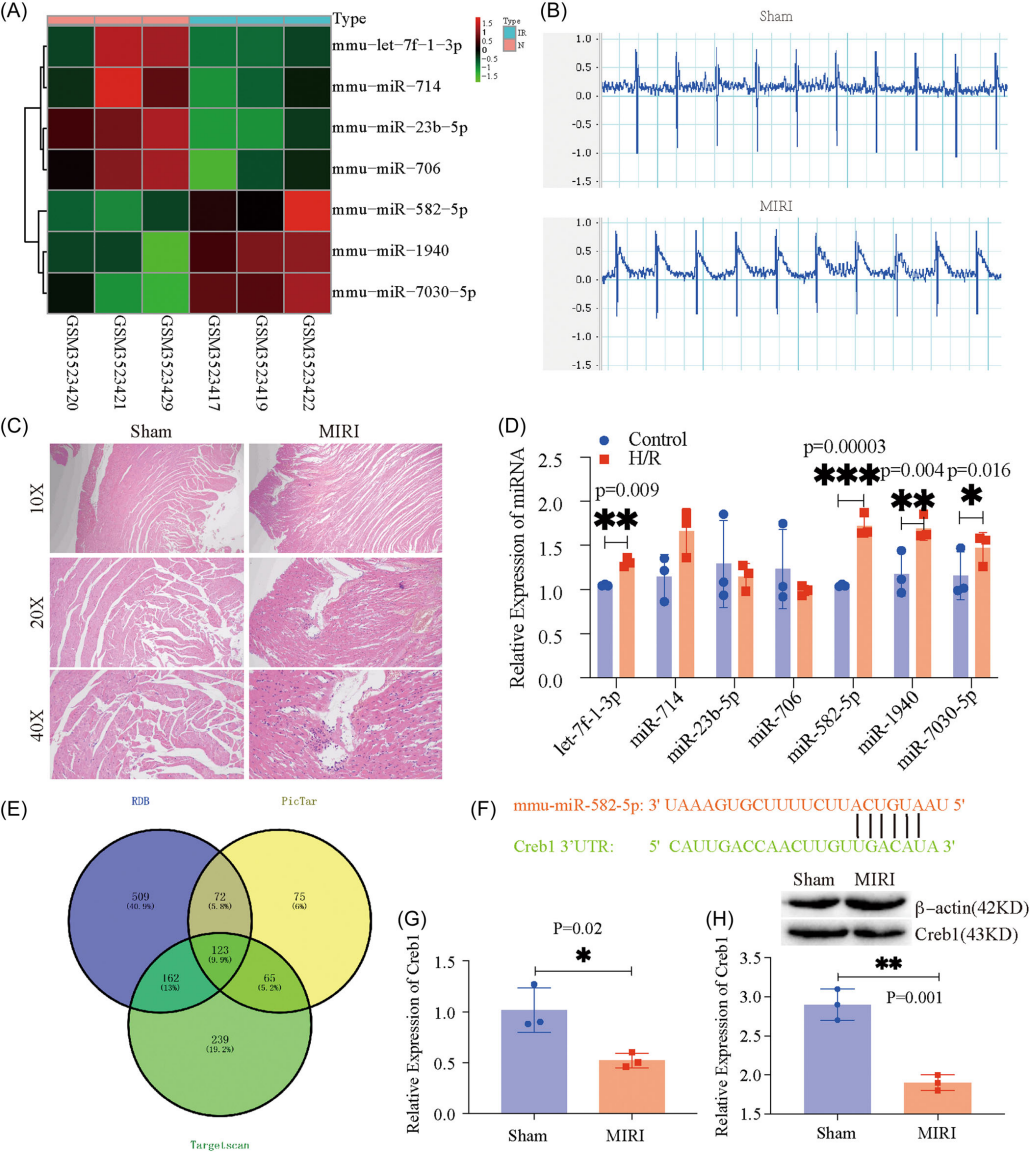
Expression of miR‐582‐5p and its target gene, Creb1, in mice MIRI models. (A) The heatmap of the gene expression of the seven significantly differentially expressed miRNAs. (B) ECG presentation in the sham and MIRI groups. (C) HE staining of myocardial tissue in the sham and MIRI groups. (D) Expression of the seven differentially expressed miRNAs in the mouse MIRI model. (E) Target gene prediction for miR‐582‐5p. (F) The binding sites between miR‐582‐5p and Creb1 were predicted on the starBase website. (G) The mRNA expression of Creb1 in the MIRI model. (H) The protein expression of Creb1 in the MIRI model. Data were expressed as the mean ± SD. 3′‐UTR, 3′‐untranslated region; Creb1, cAMP‐response element binding protein 1; ECG, electrocardiograph; HE, hematoxylin and eosin; H/R, hypoxia/reperfusion; MIRI, myocardial ischemia–reperfusion injury; miRNA, microRNA.

To search for molecular alterations associated with the occurrence and development of MIRI, we analyzed the transcriptome sequencing data of myocardial tissue from the mouse MIRI model. The bioinformatics analysis found seven significant differential miRNAs, including miR‐582‐5p, let‐7f‐1‐3p, miR‐714, miR‐23b‐5p, miR‐706, miR‐1940, and miR‐7030‐5p (Figure 1A). To validate the expression of the above differentially expressed miRNAs in cardiac tissue, we constructed a mice MIRI model. The electrocardiograph showed a significant elevation of the ST‐segment in the MIRI model mice (Figure 1B), suggesting successful model production. In addition, HE staining showed the disappearance of partial myofibers and nucleolytic, empty myofibers, interstitial edema, leaky hemorrhage, and minimal neutrophil infiltration of neutrophils in the MIRI group (Figure 1C). In addition, these identified miRNAs including miR‐582‐5p (*p* = .017), miR‐1940 (*p* = .004), and miR‐7030‐5p (*p* = .035) showed significant expression differences indicated by qRT‐PCR results (Figure 1D). By consulting the literature, miR‐582‐5p may play an important regulatory role in myocardial injury. To elucidate the regulatory mechanism of miR‐582‐5p, we further used multiple databases (miRdb, PicTar, and TargetScan) to predict the downstream target genes of miR‐582‐5p. There were 123 target genes determined in the miRdb, PicTar, and TargetScan databases (Figure 1E). In a review of the literature and experimental outcomes in the present study, Creb1 was determined as the target gene of miR‐582‐5p since mRNA 3′‐UTR region of the Creb1 gene contains a binding site of miR‐582‐5p (Figure 1F). QRT‐PCR and WB detection illustrated that the mRNA and protein expression of Creb1 was significantly decreased in the MIRI model, as compared to the normal controls (Figure 1G,H, *p* = .02 and .001, respectively). In conclusion, miR‐582‐5p may play an important regulatory role in the myocardial injury mediated by the MIRI model, possibly by regulating Creb1. However, the specific regulatory role of miR‐582‐5p/Creb1 in myocardial injury should be further investigated in subsequent experiments.

### 3.2 The expression of miR‐582‐5p was upregulated and Creb1 was downregulated in H/R‐induced cardiomyocytes

**Figure 2:**
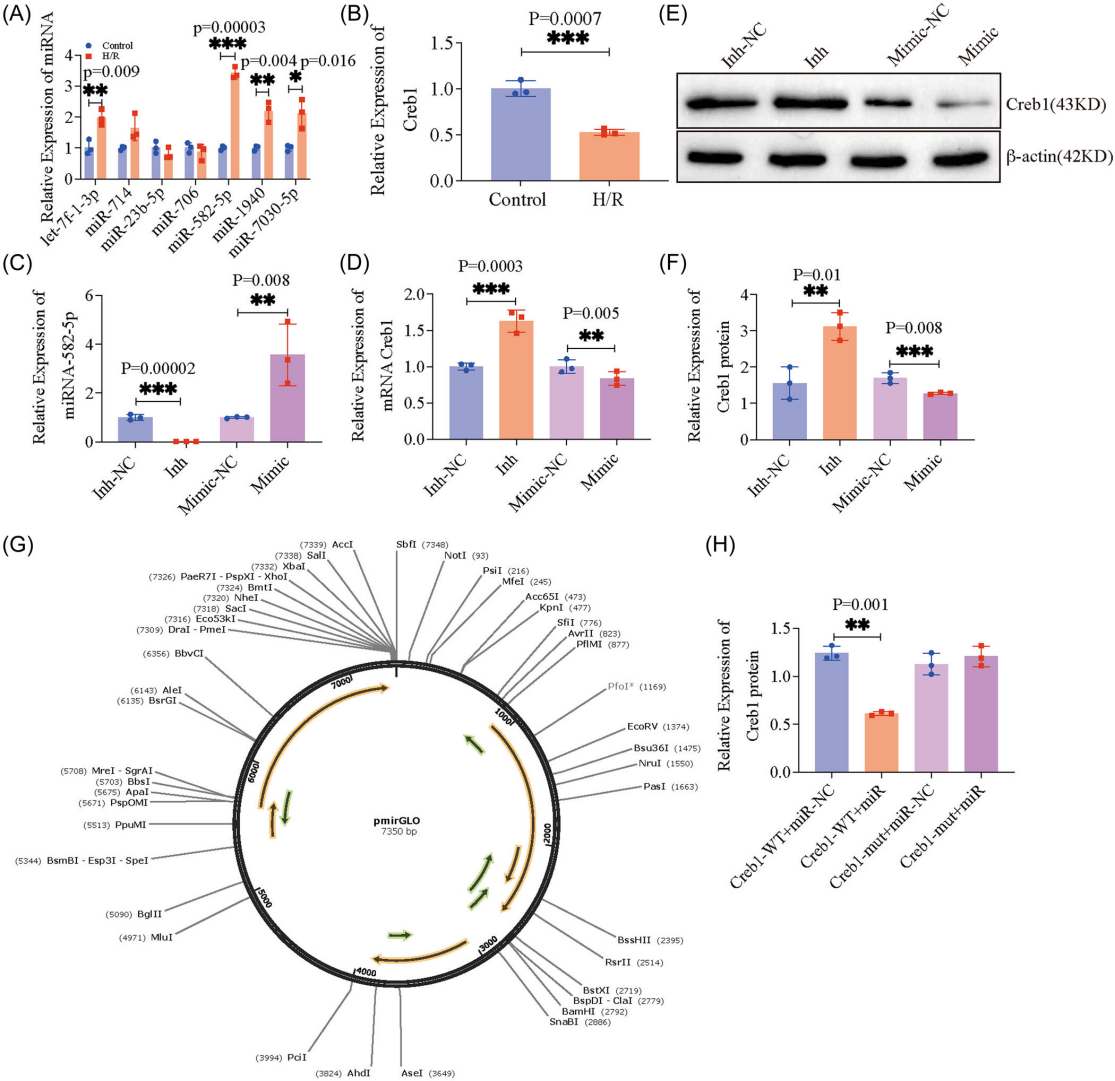
MiR‐582‐5p directly targets Creb1 in H/R‐induced cardiomyocytes. (A) Expression of the seven miRNAs in H/R‐induced cardiomyocytes. (B) Expression of Creb1 in H/R‐induced cardiomyocyte. (C) Expression of miR‐582‐5p after transfection of mimic and inhibitor of miR‐582‐5p, respectively. (D) Expression of Creb1 mRNA after transfection of mimic and inhibitor of miR‐582‐5p, respectively. (E, F) Expression of Creb1 protein after transfection of mimic and inhibitor of miR‐582‐5p, respectively. (G) Schematic representation of the pmirGLO plasmid vector. (H) Relative luciferase activity of Creb1‐Wt/Mut was examined by luciferase reporter assay in HL‐1 cells. The images are representative of three independent experiments per group and data. Creb1, cAMP‐response element binding protein 1; H/R, hypoxia/reperfusion; miRNA, microRNA; mRNA, messenger RNA; Mut, mutant; NC, negative control; Wt, wild‐type.

QRT‐PCR further demonstrated that miR‐582‐5p was distinctively increased in H/R‐induced HL‐1 cells, whereas Creb1 was significantly decreased (Figure 2A,B, *p* = .00003 and .0007 respectively). To validate the regulatory relationships between miR‐582‐5p and Creb1 in MIRI, we synthesized mimics and inhibitors of miR‐582‐5p for interference and overexpressing miR‐582‐5p in HL‐1 cells, respectively. As shown, the expression levels of miR‐582‐5p in HL‐1 cells were significantly decreased (*p* = .00002) with miR‐582‐5p inhibitor transfection and significantly increased (*p* = .008) with miR‐582‐5p mimic transfection (Figure 2C). Meanwhile, expression levels of Creb1 in HL‐1 cells were significantly increased (*p* = .0003) with anti‐miR‐582‐5p transfection and significantly decreased (*p* = .005) with miR‐582‐5p mimic transfection (Figure 2D). The expression of Creb1 protein was increased (*p* = .01) with miR‐582‐5p inhibitor transfection and significantly decreased (*p* = .008) with miR‐582‐5p mimic transfection in HL‐1 cells (Figure 2E,F). These results suggest that miR‐582‐5p may regulate Creb1 expression at the posttranscriptional level. Dual‐luciferase reports analysis demonstrated that the luciferase activity of the Creb1‐Wt + mimic group was remarkably reduced when compared to groups (Figure 2H, *p* < .01). These results indicated the direct target relationship between Creb1 and miR‐582‐5p.

### 3.3 Overexpressed Creb1‐inhibited H/R‐induced cardiomyocytes apoptosis

**Figure 3:**
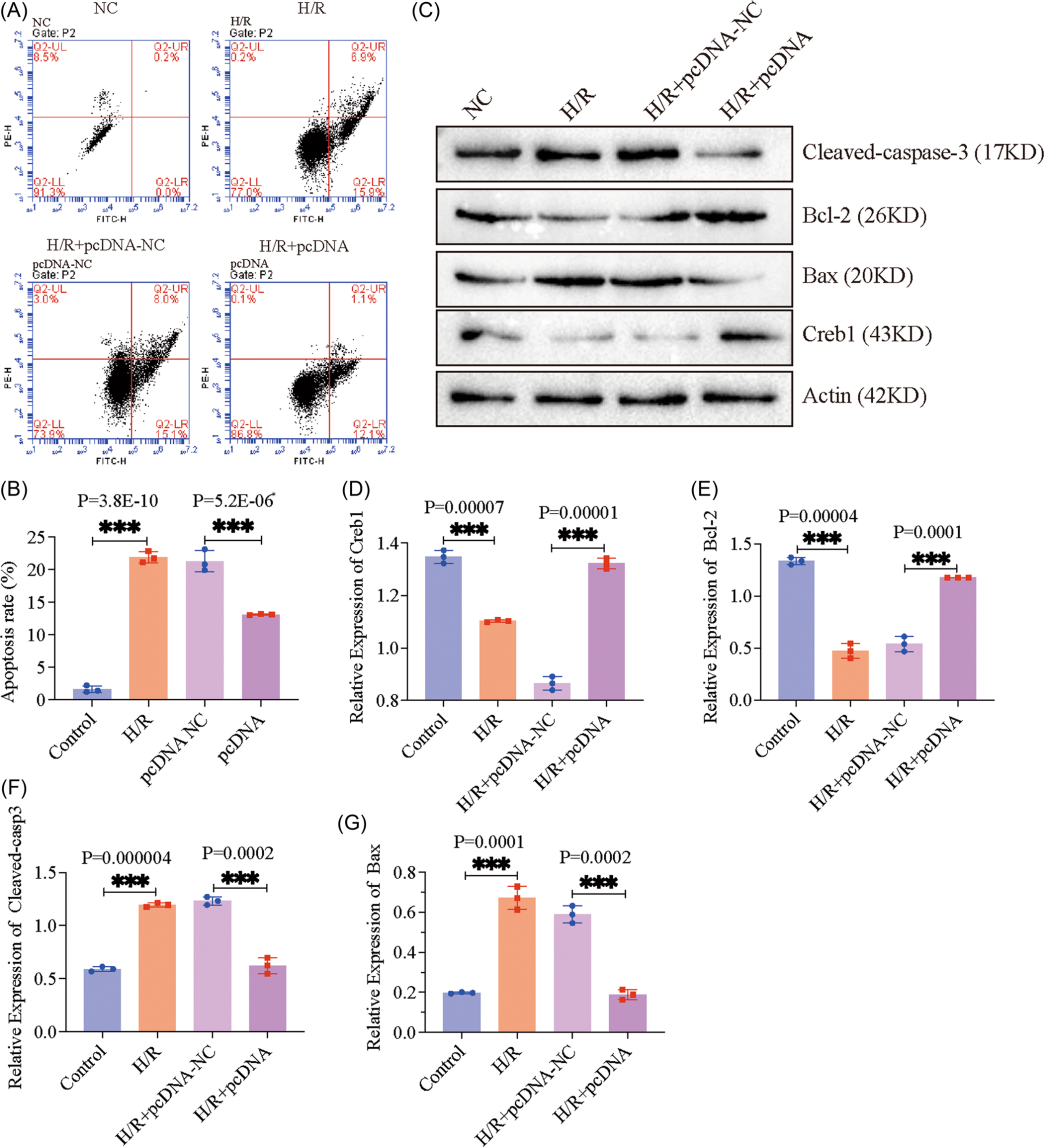
Overexpressed Creb1 inhibited H/R‐induced cardiomyocyte apoptosis. (A, B) The apoptosis rate was determined by using flow cytometry at 48 h. (C–G) Protein extracts from cells were immunoblotted with apoptosis‐related protein antibodies. β‐actin was a loading control. The quantification of Creb1 (D), Bcl‐2 (E), cleaved‐caspase 3 (F), and Bax (G) were quantified by use of FIJI software. Data are present as mean ± SD. Creb1, cAMP‐response element binding protein 1; H/R, hypoxia/reperfusion; NC, negative control.

Next, we investigated whether Creb1 expression was associated with H/R‐induced cardiomyocyte apoptosis. HL‐1 cells were transfected with pcDNA3.1‐Creb1 to overexpress Creb1, followed by H/R treatment. H/R treatment significantly induced the HL‐1 cell apoptosis, whereas Creb1 overexpression decreased the apoptosis (Figure 3A,B, *p* < .001). Then, the expression level of apoptosis‐related protein was determined by WB detection, which showed that the expression of Bax and cleaved caspase 3 were increased, and the expression of Bcl‐2 and Creb1 were decreased. However, the expression trend was partially reversed as Creb1 upregulation (Figure 3C–G, *p* < .001). These results indicated that Creb1 exerted a positive role in H/R‐induced cardiomyocyte apoptosis.

### 3.4 Downregulation of Creb1 could reverse the depression effect of miR‐582‐5p inhibitor in H/R‐induced cardiomyocytes apoptosis

**Figure 4:**
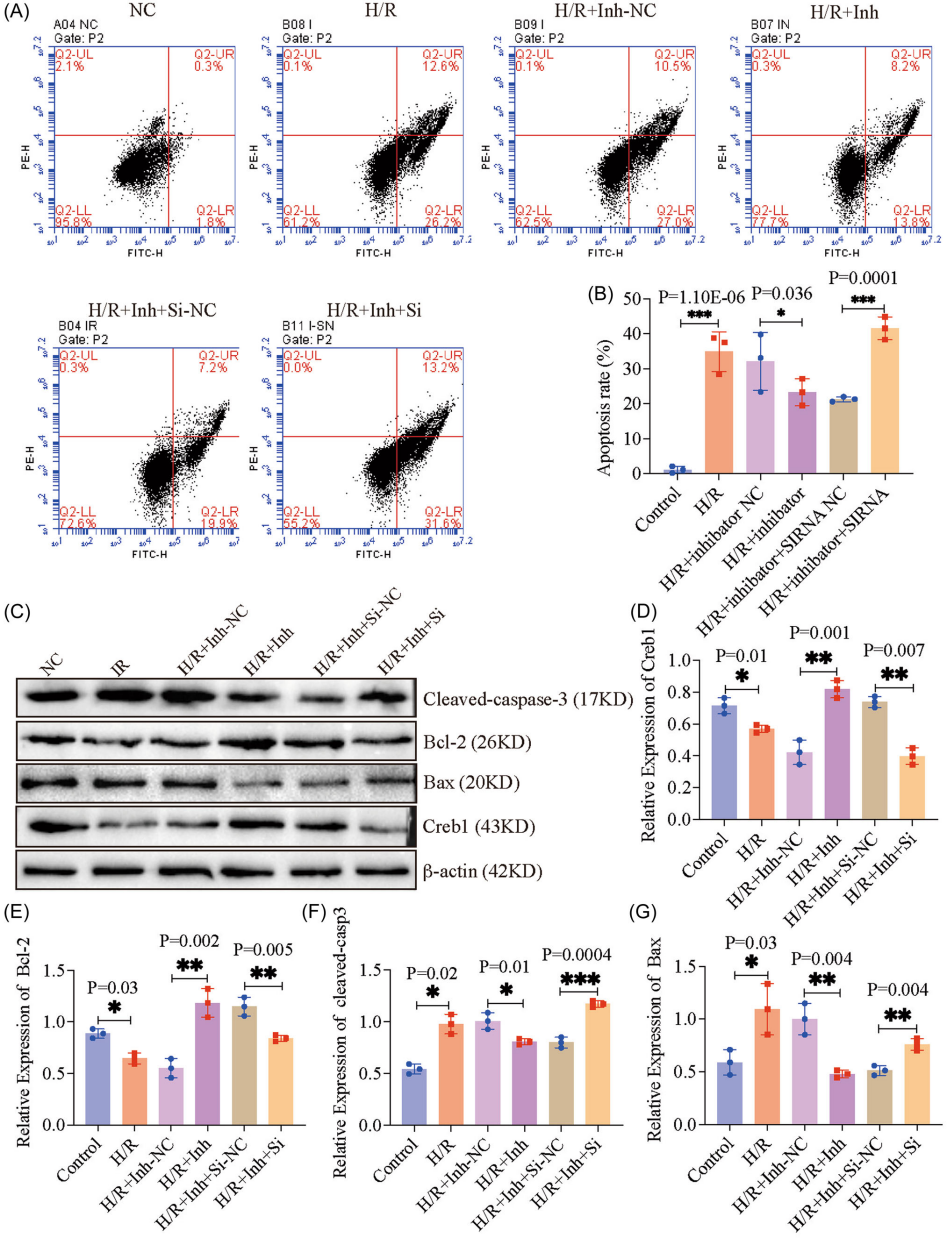
The inhibitory action of miR‐582‐5p knockdown in H/R‐induced cardiomyocytes was abolished by the downregulation of Creb1. (A, B) The apoptosis rate was determined by using flow cytometry at 48 h. (C–G) Protein extracts from cells were immunoblotted with apoptosis‐related protein antibodies. β‐actin was a loading control. The quantification of Creb1 (D), Bcl‐2 (E), cleaved‐caspase 3 (F), and Bax (G) were quantified by use of FIJI software. Data are present as mean ± SD. Creb1, cAMP‐response element binding protein 1; H/R, hypoxia/reperfusion; NC, negative control; siRNA, small interfering RNA.

To elucidate the mechanism of miR‐582‐5p in MIRI, the miR‐582‐5p inhibitor, or both miR‐582‐5p inhibitor and si‐Creb1 were transfected into H/R‐induced HL‐1 cells. Obviously, the miR‐582‐5p inhibitor suppressed the H/R‐induced apoptosis, which was significantly reversed by si‐Creb1 (Figure 4A,B, *p* < .05). The upregulation of Creb1 mediated by the miR‐582‐5p inhibitor was prominently suppressed by the presence of si‐Creb1 in HL‐1 cells under H/R treatment (Figure 4C,D, *p* < .05). Furthermore, in H/R model, the protein levels of Bcl‐2 were increased, while Bax and cleaved‐caspase 3 were decreased after transfecting miR‐582‐5p inhibitor, which was reversed in the presence of si‐Creb1 (Figure 4C,E–G, *p* < .05). These results indicated that miR‐582‐5p elicits promoting effects in H/R‐induced cell apoptosis through downregulating the expression of Creb1.

## 4 DISCUSSION

AMI‐induced MIRI is a seriously pathological process that exacerbates myocardial damage after re‐opening the blocked coronary artery.^36^ The mechanisms and prevention methods of MIRI have become a hot spot in the field of medical research.

It has been reported that the pathogenesis of MIRI is closely related to ncRNAs, including miRNAs, long noncoding RNAs, and circular RNAs.^46^, ^47^, ^48^ In the present study, by analyzing existing MIRI‐related miRNAs sequencing results from the GEO database, bioinformatics analysis revealed differential expression miRNAs in MIRI, including miR‐582‐5p, let‐7f‐1‐3p, miR‐714, miR‐23b‐5p, miR‐706, miR‐1940, and miR‐7030‐5p. Then, establishing HL‐1 cell models in vitro and mouse animal models in vivo, the results showed that miR‐582‐5p was significantly increased in cardiomyocytes after H/R. MiR‐582‐5p stimulates apoptosis and cycle arrest in multiple cancer cells and inhibits the viability, proliferation, migration, and metastasis of cancer cells.^49^, ^50^, ^51^ During the osteogenic differentiation of human bone marrow mesenchymal stem cells (hBMSCs), miR‐582‐3p can inhibit the hBMSCs osteogenic differentiation and induce apoptosis.^53^ Furthermore, our findings are consistent with the liver transplantation‐related ischemia–reperfusion injury. Studies have shown that liver stimulants (hepatic stimulator substances) can reduce CDK1 stability and translation by regulating the expression of a variety of miRNAs, such as miR‐410‐3p, miR‐490‐3p, and miR‐582‐3p, thereby protecting the liver from ischemia–reperfusion injury.^18^ However, in cerebral ischemia‐related diseases, miR‐582‐3p expression and function present opposite manifestations.^19^, ^53^ Anyway, these findings suggest that miR‐582‐5p is closely related to MIRI.

Most studies have shown that miRNAs take part in multiple biological processes in the normal state or during the occurrence of multiple diseases by regulation of downstream target genes.^5^, ^54^, ^55^, ^56^ Therefore, to elucidate the mechanism of miR‐582‐5p action in MIRI, multiple miRNA‐related target gene prediction databases were used to predict the target genes of miR‐582‐5p. Our results predicted a potential targeted binding site between miR‐582‐5p and Creb1 mRNA 3′‐UTR. Previous studies have shown that Creb1 is primarily involved in the progression and metastasis of multiple tumors,^57^, ^58^, ^59^, ^60^, ^61^ along with ischemic injury and ischemia–reperfusion injury processes in multiple tissues and organs.^22^, ^23^, ^24^, ^26^ In this study, in both HL‐1 cells and mouse animal models, we found that Creb1 was significantly downregulated in cardiomyocytes after H/R, in contrast to the expression of miR‐582‐5p, suggesting an association between miR‐582‐5p and Creb1. Subsequently, a dual‐luciferase reporter system assay revealed a clear targeted binding site between miR‐582‐5p and Creb1 mRNA 3′‐UTR, solidifying the role of the miR‐582‐5p/Creb1 axis in the pathogenesis of MIRI.

The presence of apoptosis and autophagy conditions denote the main pathological mechanisms of MIRI.^61^, ^62^, ^63^ Apoptosis involves more signal transduction pathways and cross‐regulation among each other.^46^ Recently, the role of ncRNAs in regulating apoptosis has drawn much attention. In the present study, the flow cytometry results showed that H/R significantly promoted the HL‐1 cell apoptosis. Additionally, to investigate the function of miR‐582‐5p on the apoptosis regulation by targeting the regulation of Creb1, HL‐1 cells in our study were transfected with miR‐582‐5p inhibitor alone, or cotransfected with miR‐582‐5p inhibitor and Creb1 interference fragment, followed by H/R. We found that H/R‐induced cardiomyocyte apoptosis was suppressed by miR‐582‐5p inhibitor, which was significantly raised by the Creb1 interference fragment. The upregulation of Creb1 mediated by miR‐582‐5p inhibitor was inhibited in the presence of Creb1 interference fragment in HL‐1 cells under H/R treatment. These findings indicated that miR‐582‐5p exerted an accelerative effect in H/R‐induced cardiomyocyte apoptosis depending on downregulating its target Creb1.

Apoptosis is an extremely complex process, with many genes involved in the apoptotic process, including lethal and survival genes. Members of the Bcl‐2 family, including prolife (e.g., BCL‐XL) and prodeath (e.g., BAX, BAK) proteins, play a crucial role in apoptosis regulation.^64^, ^65^, ^66^ The ratios between the two Bax/Bcl‐2 proteins were found to be a crucial factor in determining the strength of the inhibitory effect on apoptosis.^68^ Moreover, the caspase protein family plays an extremely critical role in apoptosis, and caspase‐3 represents the most critical apoptotic executive protease during apoptosis.^68^, ^69^ Our work revealed that expression of Bcl‐2 was increased, while Bax and cleaved‐caspase 3 were decreased in miR‐582‐5p inhibitor transfected cardiomyocytes under H/R treatment, which were reversed by the presence of Creb1 interference fragment. These suggested that the miR‐582‐5p/Creb1 axis‐modulated cardiomyocyte apoptosis in MIRI might be related to alteration of inflammatory factors including Bax, Bcl‐2, and cleaved‐caspse‐3.

Taken together, a series of experimental studies can preliminarily confirm that miR‐582‐5p can promote cardiomyocyte apoptosis by inhibiting the expression of Creb1 in MIRI. The mechanism underlying the promoting apoptosis function exerted by miR‐582‐5p/Creb1 axis may be related to Bax, Bcl‐2, and cleaved‐caspse‐3. However, limitations in this study include the absence of clinical data verification, since myocardial samples from clinical patients with MIRI are barely available. Therefore, the application of new and more effective models, such as the development and application of nonhuman primates and cardiac organoids, will further support our findings. Finally, the development of highly efficient and safe RNA transmission systems is also the key to achieving clinical translational therapy for miRNA‐related outcomes.

## 5 CONCLUSION

This study uncovered miR‐582‐5p promoting cardiomyocyte apoptosis in MIRI through targeting Creb1. The potential mechanism of the miR‐582‐5p/Creb1 axis in promoting cardiomyocyte apoptosis may be related to the actions of Bax, Bcl‐2, and cleaved‐caspse‐3. Hence, our findings highlight the importance of miRNA regulation and provide novel molecular mechanisms and therapeutic strategies for MIRI treatment.

## AUTHOR CONTRIBUTIONS

Xiang‐Feng Bai, Rui‐Ze Niu, and Lu‐Qiao Wang drafted the manuscript. Rui‐Ze Niu, Wei Yang, and Song Mei revised the manuscript. Song Mei, Huang Sun, and Wen‐Jie Wang performed experiments. Ke‐Xiang Feng and Dian‐Lun Qian made substantial efforts in the data verification and language polishing of the revised manuscript. Xiang‐Feng Bai, Rui‐Ze Niu, and Jie Tao designed the research and revised the manuscript. All authors checked and approved the final manuscript.

## CONFLICT OF INTEREST

The authors declare no conflict of interest.

## ETHICS STATEMENT

All experimental procedures using animals were approved by the Animal Care and Welfare Committee of Kunming Medical University with the approval number: kmmu2021487. All experiments conformed to the Guide for the Care and Use of Laboratory Animals.

## Data Availability

The datasets supporting the conclusions of this article are included within the article.
